# Easy Synthesis and Characterization of Holmium-Doped SPIONs

**DOI:** 10.3390/nano8060430

**Published:** 2018-06-13

**Authors:** Magdalena Osial, Paulina Rybicka, Marek Pękała, Grzegorz Cichowicz, Michał K. Cyrański, Paweł Krysiński

**Affiliations:** Faculty of Chemistry, University of Warsaw, Pasteura 1 Street, 02-093 Warsaw, Poland; magdalena@osial.eu (M.O.); p.rybicka@student.uw.edu.pl (P.R.); pekala@chem.uw.edu.pl (M.P.); gcichowicz@chem.uw.edu.pl (G.C.); mkc@chem.uw.edu.pl (M.K.C.)

**Keywords:** superparamagnetic iron oxide nanoparticles, SPIONs, rare-earth doping, holmium, endoradiotherapy

## Abstract

The exceptional magnetic properties of superparamagnetic iron oxide nanoparticles (SPIONs) make them promising materials for biomedical applications like hyperthermia, drug targeting and imaging. Easy preparation of SPIONs with the controllable, well-defined properties is a key factor of their practical application. In this work, we report a simple synthesis of Ho-doped SPIONs by the co-precipitation route, with controlled size, shape and magnetic properties. To investigate the influence of the ions ratio on the nanoparticles’ properties, multiple techniques were used. Powder X-ray diffraction (PXRD) confirmed the crystallographic structure, indicating formation of an Fe_3_O_4_ core doped with holmium. In addition, transmission electron microscopy (TEM) confirmed the correlation of the crystallites’ shape and size with the experimental conditions, pointing to critical holmium content around 5% for the preparation of uniformly shaped grains, while larger holmium content leads to uniaxial growth with a prism shape. Studies of the magnetic behaviour of nanoparticles show that magnetization varies with changes in the initial Ho^3+^ ions percentage during precipitation, while below 5% of Ho in doped Fe_3_O_4_ is relatively stable and sufficient for biomedicine applications. The characterization of prepared nanoparticles suggests that co-precipitation is a simple and efficient technique for the synthesis of superparamagnetic, Ho-doped SPIONs for hyperthermia application.

## 1. Introduction

In recent years, magnetic nanoparticles like magnetite (Fe_3_O_4_) have allowed for significant progress in the field of drug delivery and cancer treatment [1,2]. Efforts focused on the rapid development and study of magnetic iron-oxide-based nanoparticles have drastically increased, especially in the case of their applications in oncology [3], where highly efficient carcinogenic cell destruction is needed.

Nanosized iron oxide exhibits a wide range of magnetic properties depending on the shape, size and chemical composition of the particles. Magnetite nanoparticles ranging from a few nanometers up to 100 nm are very attractive in a wide array of modern scientific fields, such as nanotechnology [4,5,6] biotechnology [7], MRI contrast agents [8], magnetic separation and immobilization [9] etc. These nanomaterials can also be effective for medical applications including bioimaging [10] and biosensing in diagnostics (theranostics) [11,12], controlled drug delivery and cancer therapeutics [13,14,15,16]. Several excellent reviews have been published on the synthesis, functionalization and application of magnetic nanoparticles [17,18].

Much of the literature focuses on the medical application of nanoparticles based on the superparamagnetic iron oxide nanoparticles SPIONs because of their easy synthesis, stability and biocompatibility [19,20,21]. Due to their biocompatibility, they are already used in clinical trials [22,23]. A very interesting prospect is to enhance their size-dependent properties like low toxicity, facile synthesis [24], surface-to-volume ratio, superparamagnetism [25,26] and affect the magnetic interactions through doping the core of nanoparticles with different metal ions. Usually transition metals are effective dopants, changing the magnetic properties of iron oxide nanoparticles [27,28]. Whilst doping of magnetite with lanthanides such as Ho, Gd, Tb, as well as other metals, e.g., Re, Y, is common and simple in principle, establishing the synthesis procedure with controlled shape, size and magnetic properties is difficult [10,15,16]. Lanthanide-doped nanoparticles can be directed to the tumour tissues with the help of an external magnet. Thus, they can serve not only as drug carriers in targeted drug delivery or magnetic hyperthermia, but also in so-called endoradiotherapy. The latter feature can be easily implemented by replacing the “cold” atoms of lanthanides in the superparamagnetic core with their “hot” nuclei, emitting soft β(-) radiation suitable for the internal radiotherapy, localized directly within the tumour. Thus, these nanoparticles can be used simultaneously in targeted drug delivery, hyperthermia and endoradiotherapy. Moreover, the amount of radionuclides per single nanoparticle greatly exceeds the amount of radionuclides per single molecule found in radiopharmaceuticals being currently used in radiotherapies. Therefore, such multifunctional nanoparticles may become indispensable in many areas of modern medicine. For that application the lanthanides of ionic radius matching the SPIONs crystal lattice and high magnetic moment have to be chosen. The spinel structure in magnetite-based nanoparticles of formula MFe_2_O_4_, where Fe^3+^ ions occupy octahedral sites [29] and divalent metal ions M such as Fe^2+^, Ni^2+^, etc. occupy the tetrahedral positions in the crystal [30], can be modified through the substitution of the Fe^3+^ with different trivalent ions. Substitution with ions with an ionic radius similar to Fe^3+^ (0.785 Å) [31] does not affect the magnetic behaviour of the doped SPIONs significantly, while the incorporation of lanthanide ions like Ho^3+^ (1.01 Å) [32] with a large radius, ca. 30% larger than Fe^3+^, may lead to core anisotropy, crystallographic lattice alterations and a decrease in the magnetic properties due to the Fe(III) and Ho(III) ionic radii mismatch [33,34].

The influence of the incorporation of lanthanide ions into the core of SPIONs is still poorly understood. We are aware of the fact that the largest, octahedral site of the magnetite lattice can accommodate cations up to ca. 85–90 pm radius, and there is a large ionic radii mismatch for Ho^3+^ (104 pm) as compared to Fe^3+^ (78.5 pm) ions that are to be replaced in this lattice. However, the literature data [35] and our results for SPIONs modified with Tb (106 pm) [33] indicate that such replacement is possible, at least for terbium cations, requiring local lattice distortion and favouring incorporation of these ions into octahedral and surface sites. On the other hand, most ferrites are ferrimagnetic, i.e., part of magnetic moments of the constituent ions are antiparallel, partially compensating for the overall magnetic moment of SPIONs. Therefore, the introduction of lanthanide metal ions of different ionic radii than Fe^3+^ may alter the crystallographic structure, resulting in an increase of unpaired spins and enhancing the saturation magnetization. This behaviour would be very advantageous for a drug delivery system based on SPIONs.

Since the properties of magnetic nanoparticles are strongly related to their shape and size, various methods and synthetic conditions are directed towards control over their chemical composition, morphology of formed crystallites, degree of agglomeration and magnetic properties.

The literature refers to co-precipitation, the microemulsions polyol process, solvothermal and sonochemical techniques as suitable solutions for the preparation of iron-oxide-based nanoparticles dispersed in water [36,37,38]. Among the many SPIONs synthetic procedures, co-precipitation is a promising technique due to its simplicity and productivity. The conditions of synthesis like ion concentration, pH of the solution as well as duration of synthesis and heat treatment are crucial to determine the physicochemical properties of SPIONs.

In this paper, we report on the morphology and magnetic and structural properties of Ho-doped iron oxide magnetic nanoparticles synthesized by the co-precipitation method from a solution containing different molar concentrations of all ions forming the nanoparticle core. To avoid elimination by clearance organs, the hydrodynamic diameter of the nanoparticles should remain in the range of 10–100 nm [16]. Therefore, we focused on nanoparticles that were uniform in size, with a hydrodynamic diameter below 100 nm. Holmium dopant was chosen since it can be easily replaced by its ^166^Ho radionuclide emitting β(-) “soft radiation suitable for targeted endoradiotherapy” and due to its high magnetic moment (~10.6 µ_B_) [39], which is expected to enhance a magnetization of ferrites studied. On the other hand, the atomic size mismatch is found to distort the atomic lattice and weaken the magnetic properties.

## 2. Materials and Methods

Iron (II) chloride tetrahydrate FeCl_2_∙4H_2_O puriss p.a. ≥99% (RT), iron (III) chloride hexahydrate FeCl_3_∙6H_2_O Aldrich ACS reagent 97% were supplied from Sigma-Aldrich, Germany, holmium (III) chloride hexahydrate HoCl_3_∙6H_2_O 99.9% trace metals was obtained from Sigma-Aldrich, 25% ammonia solution NH_4_OH was supplied from CHEMPUR, Poland. All chemicals were of analytical grade standards and used as received. Deionized water with resistivity 18.2 MΩ cm at 25 °C was obtained using the Milli-Q ultra-pure water filtering system (Merck, Darmstadt, Germany). Successfully prepared SPIONs were modified with 3-phosphonopropionic acid obtained from Sigma-Aldrich with 94% grade acid with the use of analytical-grade NaOH from CHEMPUR, Poland.

**Figure 1:**
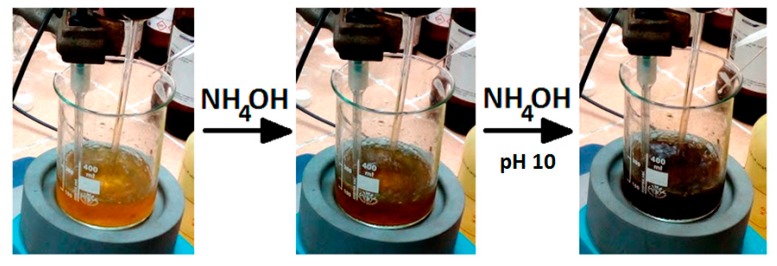
Image of the co-precipitation of Ho-doped ferrite nanoparticles under NH_3_ addition.

SPIONs were synthesized by the co-precipitation technique in aqueous media containing different batches of ions having a molar percentage of Ho(III) vs. total iron content of 1%, 2.5%, 5%, 7.5% and 10%. The required amounts of salts were dissolved in a beaker to reach the complete dissolution of 87.5 mL solution with pH around 1.9 and molarity 0.1 M of the FeCl_2_∙4H_2_O, 0.1 M of the FeCl_3_∙6H_2_O and HoCl_3_∙6H_2_O in amounts from 1–10% instead of Fe^3+^ ions. The solution was stirred mechanically for 10 min at a rate of 500 rpm (chosen as the minimum to obtain a stable vortex), and after that the ammonia was added drop by drop to the solution, and stirred for an additional 30 min. During the addition of ammonia the solution turned black, indicating the formation of a suspension with nanoparticles (see Figure 1). The schematic reaction taking place during synthesis is presented as follows:

*x*Fe^3+^ + (2 − *x*)Ho^3+^ + Fe^2+^ + 8OH^−^ → Ho_(2−*x*)_Fe*_x_*FeO_4_ + 4H_2_O.

After 30 min the beaker with suspension was left on the magnet for an additional 30 min for sedimentation, and suspended nanoparticles were washed with water and acetone alternatively to remove the residual NH_4_OH and all unreacted compounds.

Nanoparticles were stabilized with 3-phosphonopropionic acid (CEPA), where 20 mg of CEPA were used per 1 mg of nanoparticles. The SPIONs were suspended in water, and then a CEPA solution with a pH of ~5 (set by the addition of an appropriate amount of 0.1 M NaOH) was added dropwise with mechanical stirring. After CEPA addition into the SPIONs suspension, the solution was continuously stirred for the next 30 min and the suspension of nanoparticles stabilized with CEPA was washed several times with water to remove any unreacted products.

Transmission electron microscopy images were recorded using EF-TEM, Zeiss Libra 120 Plus, Stuttgart, Germany microscope operating at 120 kV. Samples were prepared from the sonicated aqueous suspensions and a drop was placed on the copper grid covered with formvar/carbon layer and dried in air. Dynamic light scattering (DLS) was used as a complementary technique to analyse the size of nanoparticles. Measurements were carried out with Malvern Instruments Zetasizer Nano ZS, Malvern, United Kingdom. The powder X-ray diffraction patterns were recorded with a powder diffraction X-ray diffractometer (PXRD) Bruker D8 Discover, Massachusets, USA operating with Debye–Scherrer geometry with Cu Kα radiation with line λ = 1.540598 Å and a scan rate of 1° per minute in 0.012° steps covering the 2θ angle range from 20° to 130°. Measurements were performed at RT. The X-ray photoelectron spectroscopy (XPS) measurements were performed using PHI 5000 VersaProbe (ULVAC-PHI) spectrometer, Gothenburg, Sweden with monochromatic Al Kα radiation (hν = 1486.6 eV) from X-ray source operating at 100 µm spot size, 25 W and 15 kV. XPS data were analysed with CasaXPS software ver. 2.3.16, Wimslow, United Kingdom. The binding energy values were calibrated and normalized to the C 1s peak at 284.8 eV. The magnetic behaviour of samples was verified with a QD vibrating sample magnetometer VSM over the magnetic field range from −2.0 T to +2.0 T in the temperature from 100 K to 300 K stabilized to accuracy of about 0.01 K. Magnetization and coercive fields were measured with accuracy better than 1%. Thermogravimetric analysis (TGA) was performed with TGA Q50 (TA Instruments), New Castle, USA, under nitrogen.

## 3. Results

SPIONs were synthesized through the co-precipitation method, which is a widely used technique for the preparation of SPIONs from aqueous solutions [40,41]. To verify how changes in the holmium doping affect the shape, size, crystallinity and magnetic properties of the synthesized SPIONs, multiple techniques were used. The magnetic properties of the superparamagnetic core are affected by size and shape. Smaller SPIONs with a high surface area to volume ratio yield smaller mass magnetization than larger SPIONs due to the increased contribution of the surface anisotropic layer decreasing the overall magnetization. This is usually explained in terms of a core-shell model, where the magnetically dead layer, strains and distortions at the surface are claimed to be responsible for the particle size effects [42]. Therefore, in order to elucidate only the effects of holmium doping on the magnetic behaviour of SPIONs, we have chosen a synthetic procedure yielding SPIONs of low size distributions and good crystallinity. The controlled size distribution is also beneficial from the point of view of their prospective medical applications, as discussed in Section 1.

### 3.1. Morphology Studies

**Figure 2:**
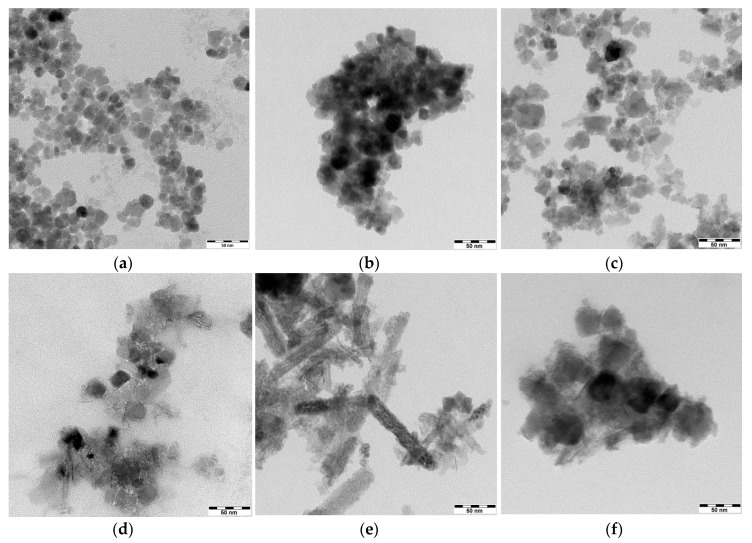
TEM images of (**a**) undoped iron oxide SPIONs, and doped with (**b**) 1%; (**c**) 2.5%; (**d**) 5%; (**e**) 7.5%; (**f**) 10% of holmium obtained by co-precipitation (scale bar: 50 nm).

**Figure 3:**
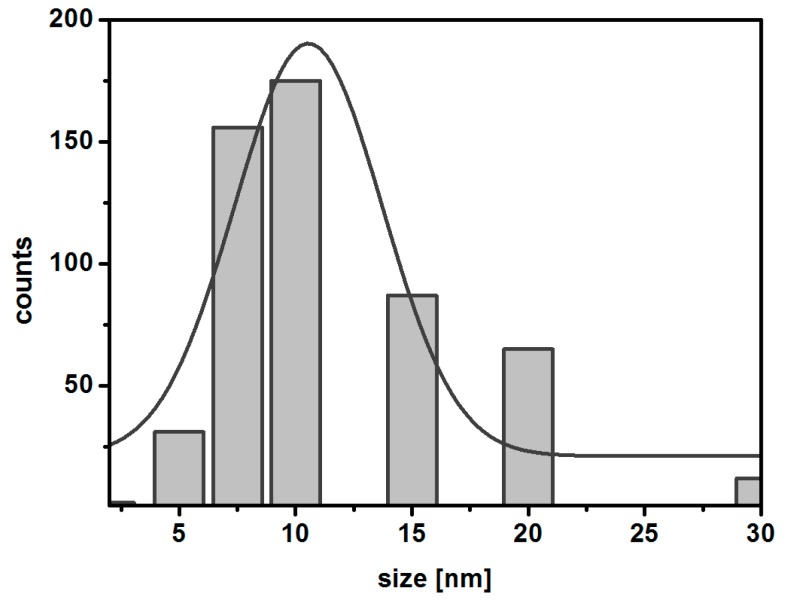
Histogram for Fe_3_O_4_@2.5%Ho.

The changes of morphology of SPIONs under Ho doping were studied with TEM. The images obtained by TEM reveal the differences in the morphology for nanoparticles with various concentrations of Ho^3+^ vs. Fe^3+^ used during the synthesis. The high level of aggregation observed on the presented images is caused by sample preparation for TEM analysis: the ethanol suspension is placed on the TEM grids and during the solvent evaporation the nanoparticles aggregate (the Marangoni effect). The literature attributes the dependence of morphology to the experimental conditions, indicating the formation of nanoparticles (SPIONs) having spherical [43], quasi-spherical [44], and even octahedral [45] shapes. As can be seen in Figure 2, the aggregates of undoped nanoparticles (a), doped with 1% (b), and 2.5% (c) of holmium have a spherical shape with an average diameter of about 10–15 mm, whereas higher doping results in an irregular axial growth of the crystals and an increase of the grain size up to 30–40 nm. Spherical particles, similar to pristine magnetite nanoparticles (Figure 2a), are formed when holmium doping does not exceed 2.5%, only slightly affecting the crystal structure of nanoferrites. Exceeding the critical value of the holmium dopant in the ferrite lattice stimulates the crystal growth in one direction. Particles shown in Figure 2a,b appear to be monodispersed, and of similar morphology. Typical histogram exemplifying the size distribution of nanoparticles for Fe_3_O_4_@2.5%Ho, is shown in Figure 3, below.

### 3.2. Size Distribution and Zeta Potential Studies

The size distribution of all samples was also investigated by means of dynamic light scattering (DLS) in an aqueous dispersion. Likewise assessed from the TEM analysis, the size of the investigated SPIONs is similar; however, SPIONs are not stabilized with any organic shell, and therefore they tend to precipitate spontaneously within a few minutes. Therefore, we stabilized them with a CEPA shell.

**Figure 4:**
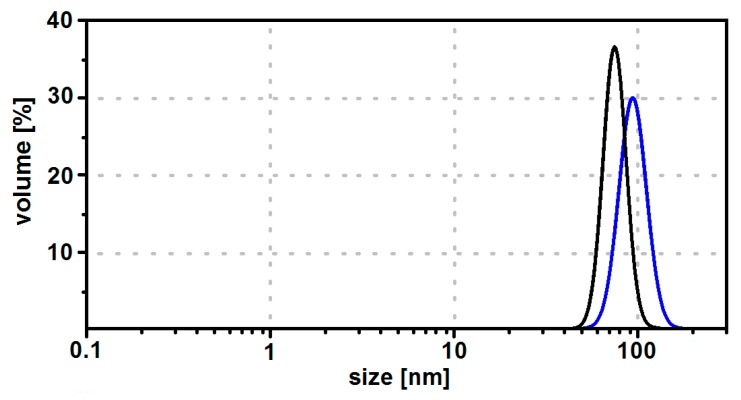
Size distribution by volume for Fe_3_O_4_@2.5%Ho unmodified (blue curve) and modified with CEPA (black curve).

As can be seen in Figure 4, the size of the unmodified SPIONs containing 2.5% of holmium is ca. 89 nm in diameter, while after modification of their surface with CEPA this value is lower by ca. 20 nm. A lower diameter of SPIONs covered with CEPA suggests that they are more dispersed than the unmodified ones. In comparison to the TEM analysis, the diameter value for uncovered SPIONs is higher because DLS reports the hydrodynamic diameter including the solvation shell of SPIONs agglomerate, whereas TEM images show only the solid core of nanoparticles. To confirm if unmodified SPIONs agglomerate, the zeta potential corresponding to the surface potential was measured. The zeta potential value for uncovered SPIONs suspended in water is about −2.7 mV, while after covering with CEPA the zeta potential value is about −32.5 mV. SPIONs covered with CEPA are stable at pH 7 for more than a week, while non-covered tend to aggregate spontaneously.

This behaviour suggests the presence of strong electric charges on the SPIONs covered with CEPA, keeping all SPIONs away from each other.

### 3.3. Thermogravimetric Analysis

**Figure 5:**
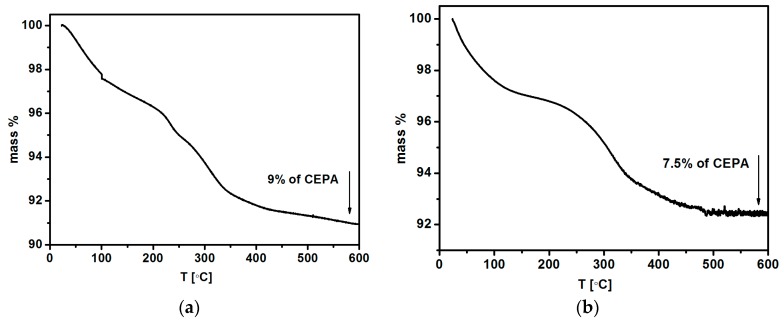
Thermograms of the SPIONs modified with 3-phosphoropropionic acid: (**a**) Fe_3_O_4_@1%Ho@CEPA, and (**b**) Fe_3_O_4_@2.5%Ho@CEPA.

The TGA analysis of nanoparticles doped with 1% and 2.5% of holmium and surface-modified with 3-phosphoropropionic acid was carried out in the range from ambient temperature to 600 °C with a heating rate of 10 °C/min under the nitrogen atmosphere. The TG curves indicate a total weight loss of Fe_3_O_4_@2.5%Ho nanoparticles due to the decomposition of the organic material from the surface of nanoparticles. On the basis of the thermograms presented in Figure 5, the quantity of 3-phosphoropropionic acid was evaluated. The organic shell constitutes ca. 7.5% for Fe_3_O_4_@1%Ho and 9% for Fe_3_O_4_@2.5%Ho of the conjugate mass, which gives 0.075 mg (4.9∙10^−7^ mol) per 1 mg of Fe_3_O_4_@1%Ho SPIONs, and 0.09 mg (5.8∙10^−7^ mol) per 1 mg of Fe_3_O_4_@2.5%Ho SPIONs. Based on the zeta potential studies, it is seen that such an amount of CEPA is sufficient to stabilize SPIONs.

### 3.4. Crystallographic Structure

**Figure 6:**
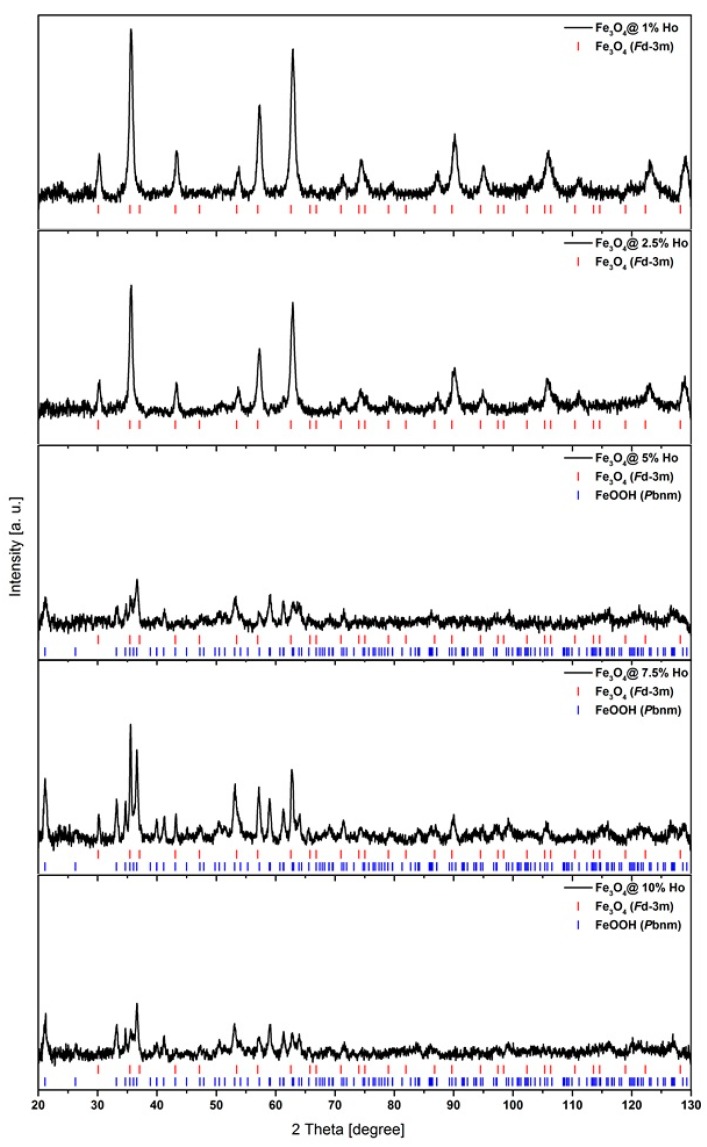
PXRD patterns of undoped and Ho-doped nanoferrite crystallites.

Powder diffraction patterns were recorded with a powder diffraction X-ray diffractometer (PXRD) operating with Debye–Scherrer geometry and Cu Kα radiation with line λ = 1.540598 Å. Measurements were performed at RT and a scan rate of 1° per minute in 0.012° steps, covering the 2θ angle range from 20° to 130°. For nanoparticles containing 1–10 at. % of holmium, the diffraction patterns consist of a series of peaks, whose positions reveal the Fe_3_O_4_ phase (*JCPDS* file, No. 19-0629 for magnetite) (see Figure 6). Due to the high iron content in the crystals, the X-ray fluorescence is revealed as a noisy background in all recorded patterns. Synthesized nanoparticles have a high surface-to-volume ratio, crystal strains and surface defects, which contributes to the full width at half maximum (FWHM) of the reflexes. Their small size also correlates to the relatively broad shape of these patterns. According to the literature, the experimental conditions influence the crystal structure of prepared nanoparticles as well as other properties [46].

Samples doped with 1 at. % and 2.5 at. % holmium reveal reflexes characteristic of the *F*d-3 m space group with single phase composition, while increasing holmium doping leads to poorer crystallinity and the additional formation of *P*bnm space group of goethite: α-FeOOH. The PXRD patterns explain the sudden change of crystal shape presented in the TEM images (see Figure 2), where crystallites with irregular shape appear. Line broadening in the recorded XRD patterns enables the calculation of the crystal size based on the Scherrer formula [47] and subsequent comparison to the TEM results. The average crystallite size in pure Fe_3_O_4_ is about 10 nm, while the size of the doped SPIONs increases to ca. 17 ± 2 nm (below 5% doping), which correlates with the TEM studies. The appearance of goethite with a 5–10% doping level leads to the poorer crystallinity and increasing amorphism of the obtained precipitate. Additionally, the shape of SPIONs is elongated due to the presence of FeOOH, which appears within this doping in the crystallites and, contrary to Fe_3_O_4_, crystallizes as a rhombic structure. [48]. In accordance with the literature [39], we do not observe a separate phase of Ho_2_O_3_.

The literature [35] suggests that lanthanide ions are incorporated into the octahedral sites of the crystallographic lattice, while such a replacement of Fe^3+^ with larger ions can cause a distortion of the lattice. Within this work we replaced Fe^3+^ having an ionic radius about 78.5 p, with Ho^3+^ ions, which are around 30% larger (~101 pm). The diffraction patterns show that the crystallinity is poorer for samples with the highest holmium content. Doping above 5% leads to polycrystallinity, and randomly distributed vacancies in the spinel structure appear. Drastic lattice deformation occurs with 5% holmium doping at Fe_3_O_4_ crystal. It is obvious that within such changes of the structure, the morphology of formed nanoparticles and magnetic properties should also change. We are inclined to assign the observed changes to the fact that at this doping level the amount of Ho is too large to be accommodated within the *F*d-3m space group. As a consequence, this may lead to the observed polycrystallinity of SPIONs. Nevertheless, these results show that, up to a given doping level, Ho^3+^ ions can be incorporated within magnetite crystallites and not merely adsorb onto the surface of SPIONs. Such adsorption will not lead to lattice distortions. However, we cannot exclude at least partial adsorption on Ho^3+^ ions onto a well-developed surface of SPIONs. 

### 3.5. XPS Analysis

**Figure 7:**
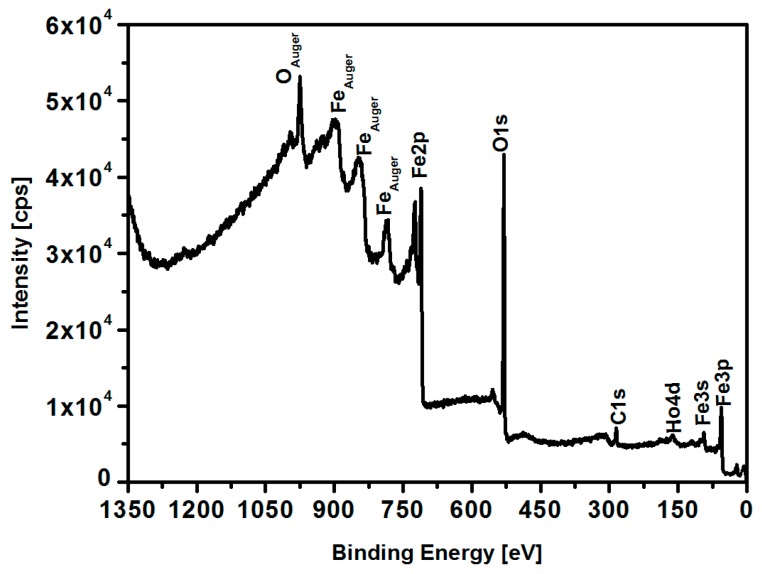
XPS survey spectrum of Fe_3_O_4_@2.5 at. % Ho nanoparticles.

In addition to the XRD studies, X-ray photoelectron spectroscopy was applied to investigate the chemical composition of the formed nanoparticles with 2.5% holmium doping. For this purpose, the analysis of the binding energy of Ho 4d and Fe 2p regions was carried out. The overall survey spectrum of the holmium-doped iron oxide nanoparticles is presented in Figure 7.

**Figure 8:**
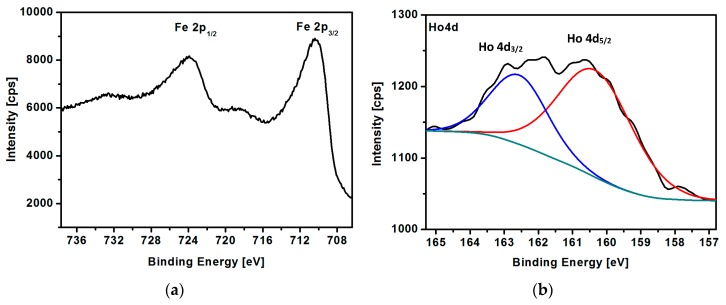
XPS spectra of (**a**) Fe 2p and (**b**) Ho 4d region for Fe_3_O_4_@2.5%Ho SPIONs.

Figure 8a,b shows the indicative regions of Fe 2p and Ho 4d binding energies, respectively, with deconvolution. As can be seen in Figure 8a, the spectrum can be fit to the doped iron oxide, where the peak positions of the Fe 2p_3/2_ and Fe 2p_1/2_ corresponding to the Fe^3+^ octahedral species [49] are located at binding energy 710.4 eV and 723.9 eV, respectively. The peak at 710.4 eV can be deconvoluted into two peaks: Fe^2+^ 2p_3/2_ and Fe^3+^2p_3/2_, respectively, ascribed to magnetite, but the precise deconvolution depends largely upon the model used [50]. The presence of satellite peaks at 718.7 eV and 732.5 eV may originate from the high-spin Fe^2+^ 2p_3/2_ shake-up. [50].

The experimental results are similar to the literature data for iron at different oxidation states [51]. Figure 8b presents the peaks at binding energies 160.5 eV and 162.7 eV, characteristic to Ho^3+^ 4d_5/2/_ and 4d_3/2_, indicating that holmium has been incorporated into the crystal lattice of iron oxide [52]. Due to the low amount of holmium in our samples, these peaks are of low intensity; therefore, their further analysis can be inaccurate.

### 3.6. Magnetic Analysis

**Figure 9:**
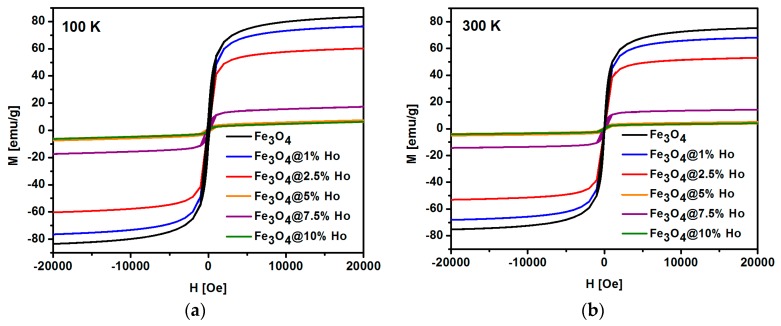
Magnetization isotherms for SPIONs with different Ho content measured at (**a**) 100 K and (**b**) 300 K.

Magnetic measurements of the ferrite-based nanoparticles were performed in a broad range of the applied magnetic field. Magnetization of the SPIONs studied increases abruptly in magnetic fields up to about 300 Oe and then approaches saturation at both 100 K and 300 K (Figure 9). The corresponding values of magnetization are lower for the 300 K isotherm than for the 100 K one. This magnetization decrease is consistent with a magnetization description given by the Brillouin function, which predicts magnetization suppression caused by the rising temperature [42,53,54]. The saturation magnetization M_s_ measured at 100 K for the pure Fe_3_O_4_ nanoparticles is found to be 83 emu/g, which is close to the value obtained for the undoped bulk magnetite. The magnetization value of nanoparticles depends on the experimental conditions during synthesis [54,55]. The M_s_ magnetization decreases down to 65 emu/g with the rise in temperature from 100 to 300 K (see Figure 9). The magnetization at both 100 K and 300 K decreases gradually when Ho doping rises from 1% to 10%. For 1% of Ho doping, only a slight M_s_ reduction is observed. The saturation magnetization, M_s_, of SPIONs doped with 2.5% being about 50 emu/g at 300 K is sufficient for magnetic separation under the applied external magnetic field (see Figure 9); however, subsequent doping above 2.5% of holmium leads to a decrease in the magnetization and degradation of the superparamagnetic properties, which is not satisfactory from a biomedical point of view.

**Figure 10:**
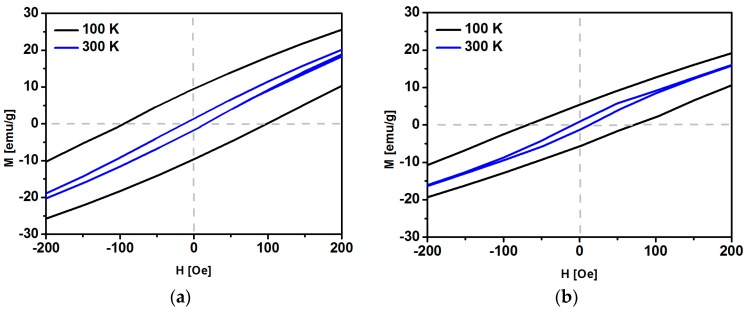
Magnetization loops K for (**a**) Fe_3_O_4_, (**b**) Fe_3_O_4_@1% Ho registered at 100 K and 300 K from −200 Oe to 200 Oe.

**Figure 11:**
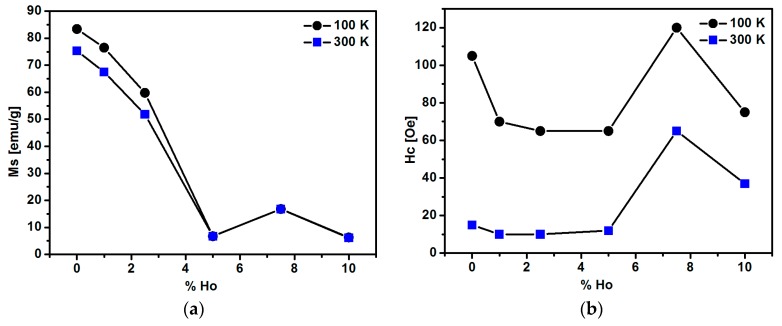
Values of (**a**) saturation magnetization at 20.000 Oe and (**b**) coercive field as a function of holmium content in SPIONs measured at 100 K and 300 K. Error bars are smaller than the data symbols.

The more detailed inspection of magnetization hysteresis loops in weak fields (Figure 10) allows us to determine the coercive field H_c_. At a temperature of 300 K, the coercive fields are equal 15 Oe and 10 Oe for the pure magnetite and SPIONs with 1% of Ho, respectively. Such a low coercive field (H_c_) as well as the shape of the magnetization curve are the characteristic features of superparamagnetic materials. The coercive field also remains low for the 2.5% and 5% doping, whereas H_c_ increases for 7.5% and 10% doping (Figure 11). On the other hand, one may notice the remarkably larger H_c_ values ranging between about 60 and 120 Oe, when the SPIONs studied become ferromagnetic at 100 K (Figure 11) [56].

A depletion of magnetic properties is observed for the higher holmium content. The saturation magnetization M_S_ drops when Ho doping is above 2.5% (Figure 11). This is accompanied by an increase in the coercive field H_c_ above 5% of Ho. The observed changes may be due to several reasons. The crystallographic lattice is found to be distorted at an Ho content exceeding 2.5%, as discussed in chapter 3.4. Crystallographic Structure.

It is also observed that the grains start to deviate from a spherical shape. Moreover, the doped ferrites become multiphasic as the additional α-FeOOH goethite phase appears in the NPs. This goethite phase is known to contain vacancies, which enhance the coercivity fields [57]. Thus, the magnetization results correlate well with the crystallographic data (vide supra).

The observed degradation of magnetic properties of studied ferrites is consistent with a two-phase approach of the so-called core-shell model, which is commonly used in analysis of fine structured materials [54]. The magnetic and electrical properties of transition-metal-based oxides are known to be strongly affected by various types of structural and chemical disorder [54,58,59,60]. The level of disorder may be accounted for by the volume fraction of disordered phase contained in the solid. In the polycrystalline and nanocrystalline solids a mean size of grains/crystallites is on the order of micrometers or tens of nanometers, respectively.

The surface layer/shell of grains and crystallites contains a material that is strongly structurally disordered as compared to the inner core of crystallites, which preserves the long-range translational symmetry characteristic of the bulk material. The thickness of the surface layer/shell extends from one to about a few nanometers and weakly depends on preparation method [43]. Thus, the volume fraction of a surface shell is known to increase abruptly when grain sizes are reduced down to the nanometer range [48,49,54]. In a rough approximation, the surface shell is often assumed to be nonmagnetic or weakly magnetic. Therefore, the magnetization of a nanostructured solid becomes suppressed as compared to the bulk material of the same chemical composition. In order to enhance their magnetization, materials like magnetite are doped with e.g., rare earth ions bearing a large magnetic moment. This approach may be successful to some extent since the atomic size mismatch between Fe and rare earth atoms starts to distort the atomic lattice and deplete the magnetic parameters. Finally, the magnetic properties of the doped ferrites studied in this work result from two competing factors: the atomic size mismatch and the high magnetic moment of Ho ions.

**Figure 12:**
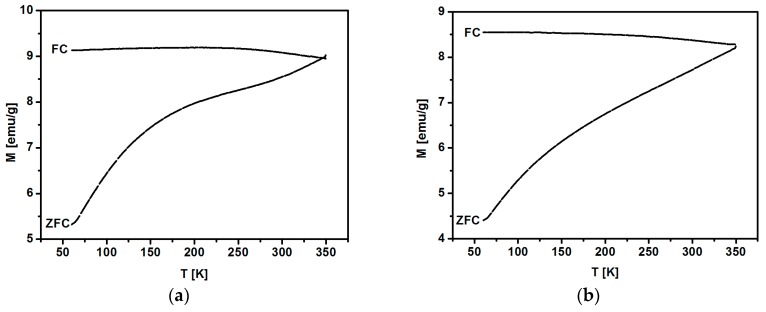
Temperature dependence of ZFC and FC magnetization at 100 Oe for nanoparticles doped with (**a**) 1% and (**b**) 2.5% of holmium.

The magnetic properties of prepared SPIONs are stable. The dependence of magnetization in the Zero-Field-Cooled (ZFC) and Field-Cooled (FC) modes in the applied DC field of 100 Oe was measured versus temperature range from 60 to 350 K (Figure 12). The FC magnetization varies only weakly in the temperature range studied. The ZFC magnetization is a monotonically increasing function of temperature; however, the ZFC maximum related to the blocking temperature T_B,_ may be located above 350 K. The shape of the ZFC curve may be due to the relatively broad grain size distribution.

## 4. Discussion

The aim of this work was to introduce a simple approach to incorporate the lanthanide, Ho^3+^, ions into the ferrite oxide nanoparticles through the co-precipitation synthesis at room temperature, forming Ho-doped multifunctional SPIONs for their further modifications for biomedical applications. SPIONs doped with holmium were successfully synthesized in conditions enabling us to control their properties, such as shape, size, magnetization and structure. We have found that 2.5% molar of holmium in the ferrite-based SPIONs is maximal value yielding SPIONs with homogeneous phase and high magnetization above 60 emu/g. Undoped and 1–2.5% Ho-doped samples have a size ranging from a few to 20 nm in diameters, whereas further increase in the holmium content causes the growth of their size and irregular shape of the grains. We also observed an unusual drop in the magnetic properties at 5% of holmium in SPIONs, which we are inclined to assign to the size mismatch of Ho^3+^ and Fe^3+^ ions, leading to crystal lattice strain, defects in the structure and the formation of the goethite phase. The conclusion on the formation of α-FeOOH in the synthesized nanoparticles is additionally supported by the appearance of coercivity under the influence of an external magnetic field. XPS analysis confirmed the presence of holmium-doped iron oxide SPIONs. In contrast to the heterogeneous samples, SPIONs doped with 1–2.5% reveal superparamagnetic behaviour at room temperature and XRD patterns confirm that they maintain a homogeneous phase with well-formed crystallites. As they are synthesized, SPIONs tend to aggregate in the aqueous media. Subsequent modification with a CEPA organic shell stabilizes the suspension due to the electrostatic repulsion of negatively charged carboxylic groups of 3-phosphoropropionic acid shell. Further studies will attempt to implement the replacement of “cold” atoms of Ho in the superparamagnetic core with its ^166^Ho radionuclide, emitting “soft” β(-) radiation limited only to the distance of several millimeters, for the multiplied therapeutic effect of endoradiotherapy and magnetic hyperthermia.

## 5. Conclusions

In summary, using an efficient co-precipitation technique we obtained and characterized SPIONs doped with holmium. Taking into account their physicochemical properties, the superparamagnetic SPIONs doped with 1–2.5% holmium can be used for biomedical applications. The incorporation of such an amount of lanthanide is optimal for maintaining high magnetization and the homogeneous crystal structure of SPIONs, as a core SPIONs for modification with organic linkers and antitumor drugs. SPIONs prepared via the proposed route have great potential application in the biomedical field.

## 6. Patents

Application no. P-424901.
